# Chicago Medical Society

**Published:** 1866-10

**Authors:** 


### PROCEEDINGS OF MEDICAL SOCIETIES.

### CHICAGO MEDICAL SOCIETY.

### SANITARY CONDITION OF THE CITY.

Prof. Davis read the sanitary report, of which he had given a verbal synopsis at a previous meeting. He stated farther, that, while during the month of June the mortality had been but 319, of which only 36 were from diseases of the bowels, during the month of July it had reached the large number of 706, of which 286 were from diseases of the bowels.

In the mortality returns at the health office, 68 deaths were reported as resulting from measles. Undoubtedly fully one-half of these were from affections of the bowels, thus making a total of 320 deaths from this class of diseases in the month of July. Dr. Davis stated, from observation in general practice during July, he could have foretold that the mortality would be very much larger during this month than it was in June. This increased mortality was undoubtedly dependent, in a great measure, upon the continued warm and sultry condition of the atmosphere.

The first cases presenting many of the symptoms of true cholera, without, however, excessive and rapid tendency to collapse, were observed on the 8th, 9th and 11th of July.

All the cases of cholera, almost without exception, had been either in portions of the city where the sanitary condition was bad, or in individuals evidently imprudent in the use of liquor, sleeping in sultry, ill-lighted and ill-ventilated apartments.

[It may be of interest to our readers to state, that during the month of August the mortality in Chicago reached the number of 940 against that of 319 for June, 706 for July, of this year, and 466 for August, 1865. Of this number, including 34 reported as deaths from teething, 516 were from diseases of the bowels; 578 were under the age of five years. There were 134 deaths from cholera and 15 from cholera morbus.—Eds.]

### OPIUM IN CHOLERA INFANTUM.

Dr. Fitch had depended for several years upon minute doses of morphine, (gr. $\frac{1}{70}$—$\frac{1}{80}$) calomel (gr. $\frac{1}{4}$—$\frac{1}{2}$) and sugar in cholera infantum with vomiting and emaciation. He had employed these remedies with most excellent results. The intervals between the doses were determined by the effects of the morphine.

Dr. Davis had found the following formula highly beneficial in many of these cases, especially in relieving vomiting:

℞ Aquæ distil. ℥ii., acet. morphiæ, gr. i., acet. plumbi, gr. xv., acid. acet. gtt. x. M. S. Dose, ten drops every three to four hours.

Dr. Paoli alluded to the very great danger of administering any preparation of opium to very young infants; he believed many children were destroyed by its injudicious use.

The remarks of these gentlemen induced a very interesting discussion upon the use of opium in young infants. It was the general opinion of the members that opium was perfectly safe for infants, however young, provided the necessary care was taken in reference to the quantity in the doses and the intervals between them. The doses should be very minute and repeated only as the symptoms indicated. In connection with opium, calomel and diuretics were considered important by some members.

Dr. Davis believed opium was exceedingly dangerous in diseases of the lungs in young children; with due caution, it was perfectly safe in almost all other diseases.

NEW ANÆSTHETIC.

Dr. B. F. Gilman, introduced as late Surgeon of the U. S. Army, presented several bottles of a new anæsthetic, with a circular describing its properties.

He claimed for it all the advantages of chloroform, ether or nitrous oxide, without danger of producing nausea, vomiting or headache. The agent was a compound of meconic acid, kinic acid, codeia and thein, dissolved in alcohol and formyl.

The remarks of Dr. Gilman and his circular gave rise to considerable discussion. Objection was expressed to the name of "Narcotina," which he applied to this new agent, as being a term already given to one of the active principles of opium. Objection was also made to the following phrases in the circular: Narcotina "is an almost instantaneous cure (?) for headache and neuralgia." "*It is free from all danger, and may safely be used by children and adults.*" Doubt was felt how far the following was absolutely correct: "It has been thoroughly tested in hundreds of cases, and is used in the U. S. Army, in hospitals in New York, Philadelphia and Boston." The question was asked to what extent codeia, thein and such agents would be vaporized and enter the lungs, by inhaling their solutions, poured on linen or sponge.

On motion, the subject was referred to a committee, consisting of Drs. Bogue, Bevan, Lyman, of Cook County Hospital, and Holmes, with the request that the committee should make use of this anæsthetic in such patients as they might find convenient, and report at some future meeting regarding its efficacy.

### ACUTE CONJUNCTIVITIS.

Dr. Holmes called the attention of the Society to the prevalence of acute conjunctivitis in the city during the past three weeks. He had been called upon to treat during this time a much larger number of cases than during the same period before in ten years. Several cases had been reported to him by medical friends. There seemed to be a popular opinion, in different sections of the city, that the disease was very prevalent.

The subject was of interest, since the testimony of our most experienced physicians would indicate that Chicago had not, for many years at least, suffered from an epidemic of conjunctivitis, as sometimes prevails in other parts of Illinois and the adjoining States. This disease prevails to such an extent in some country districts, that physicians in full general practice report, that occasionally more than a third of their patients are affected with inflammation of the conjunctiva. It would seem remarkable, in a city of 200,000 inhabitants, in a climate subject to such great and sudden changes as are experienced here, that acute conjunctivitis should be comparatively so rare.

Of the nineteen cases, observed during the past twenty days, eight were characterized by much swelling of the lids and ocular conjunctiva, and in three of these cases by considerable ecchymosis, as if small vessels had been ruptured. In five of these cases there was excessive mucopurulent discharge. In the remaining cases there was quite acute inflammation with much redness and more or less discharge, without, however, special symptoms. These cases were distributed nearly equally in the different divisions of the city, and could not be traced to special causes as regards location, habits of life, or health of the patients.

The local treatment in five of the milder cases was limited to a collyrium of alum, gr. x., and morph. sulph. gr. ii., to the ounce of rose water, instilled between the lids two or three times daily, with frequent application of tepid water to the lids.

The severe cases were all treated with solution of nitrate of silver, varying in strength from gr. x. to ℈ i. to the ounce of distilled water, applied once, and in the severest cases, twice, daily to the palpebral conjunctiva thoroughly exposed, the mucus and moisture being removed by a bit of linen. The application was made by means of a camel's hair-pencil, in such a way that the solution was not allowed to flow over the globe.

In three cases firm pressure with compresses, held in place upon the eyes by means of bands bound tightly around the head, was employed.

Special care was taken even in the mild cases that the patients should avoid all causes of irritation. Diuretics were administered, and when the condition of the patients admitted, free diaphoresis was induced by warm drinks, the patients being covered with thick blankets during the night. In two cases where there was considerable pain, nearly a grain of morphine was administered at night.

The results of this treatment were most satisfactory except in one case of muco-purulent, and two cases of mild catarrhal inflammation; in the former case an extensive slough of the upper portion of the cornea produced a large ulcer, which assumed the form of partial pannus; the lids became extensively "granulated." In the other two cases the inflammation, though slight, is still obstinate, and in spite of treatment, is becoming chronic, with hypertrophy of the conjunctiva.

### PRESSURE IN CONJUNCTIVITIS.

In reply to a question asked by a member in reference to the acknowledged value of pressure in conjunctivitis, Dr. Holmes stated that the subject was scarcely mentioned by any of the standard authorities on diseases of the eye. In a work based upon the clinical lectures of Velpeau, published nearly thirty years ago, a simple allusion is made to the fact that pressure was habitually employed by the natives of the "East" in conjunctivitis. Velpeau himself and Piorry are said to have used it thirty years since. This mode of treatment, however, seems to have been almost wholly forgotten in Paris, and to have attracted little attention among the German and English ophthalmologists.

There is a short account of its use in Cooper's Surgical Dictionary, at the close of the article "Ophthalmy," in which it is stated that Dr. Sewell, of Washington, D. C., acquired considerable reputation thirty years ago by his success in the treatment of conjunctivitis by certain applications to the mucous membrane, followed by pressure on the lids.

Stellwag of Vienna, in his valuable work on diseases of the eye, published in 1861, devotes a page to this subject; he seems to confine its use principally to conjunctivitis in children.

Dr. Holmes stated that he had employed pressure in 1862, at the personal suggestion of Stellwag, but had not found sufficient benefit, in the three cases in which he applied it, to induce him to continue its use. In the case of slough of the cornea above mentioned, pressure was carefully applied.

Subsequently, however, he had employed it in quite a number of cases, and believed it would be found sometimes of considerable advantage, if applied in the very *earliest stages* of severe inflammation of the conjunctiva. He had been disappointed in its results in advanced stages. Desmarres and others seem to have placed confidence in it; while many others subsequently found reason to discard it.

In certain cases of acute conjunctivitis, it has been found utterly impossible to apply pressure, in consequence of the great tenderness and pain in and about the eye.

More recent observations in the journals of this country and Germany seem to show that little value can be attached to the method.

